# Web-Based Interventions for Depression in Individuals with Diabetes: Review and Discussion

**DOI:** 10.2196/diabetes.9694

**Published:** 2018-09-14

**Authors:** Pamela Franco, Ana María Gallardo, Xavier Urtubey

**Affiliations:** 1 Accuhealth Santiago Chile; 2 Department of Psychology Universidad del Desarrollo Santiago Chile; 3 Department of Psychology Pontificia Universidad Católica de Chile Santiago Chile

**Keywords:** Web-based intervention, internet, depression, diabetes, cognitive behavioral therapy

## Abstract

**Background:**

Depression is twice as common in people with diabetes, and this comorbidity worsens the course of both pathologies. In clinical practice guidelines, screening and treatment of depression in patients with diabetes are highly recommended. However, depression is still both underrecognized and undertreated. To find ways to enhance their reach, psychological treatments have taken advantage of benefits of internet and technological devices as delivery formats, providing interventions that require considerably less (or even no) interaction time with therapists. Web-based treatments hold promise for effective interventions at low cost with positive results.

**Objective:**

The objectives of this review were to describe Web-based interventions for depression in individuals with diabetes and to discuss these studies’ procedures and findings in light of evidence from a wider range of interventions for depression and diabetes.

**Methods:**

A comprehensive literature search was conducted in PsycINFO and MEDLINE electronic databases. Studies were included when they met the following selection criteria: the study was available in a peer-reviewed journal mainly publishing studies written in either English or Spanish; the studied sample comprised individuals with diabetes; the intervention targeted depression symptomatology; the intervention was accessible via the internet; and the intervention was accessible via the internet with little or no clinician support.

**Results:**

Overall, 5 research studies were identified in the review. All studies were randomized controlled trials, and most used a wait list as a control; 4 studies reported treatment dropout, rates of which varied from 13% to 42%. Studies supported the notion that the Web-based format is a suitable psychology service delivery option for diabetic individuals with depression (effect size range for completers 0.7-0.89). Interventions varied in their characteristics but most were clinical-assisted, had a cognitive behavioral therapy approach, used diabetes-specific topics, had a weekly modular display, used homework assignments, and had some adherence management strategy. These characteristics are consistent with the intervention features associated with positive results in the literature.

**Conclusions:**

The analyzed studies’ findings and procedures are discussed in light of evidence drawn from a wider range of reviews on Web-based interventions for depression and diabetes. Consistent with previous research on depression treatment, Web-based interventions for depression among individuals with diabetes have shown positive results. Future research should contribute new evidence as to why these interventions are effective, for whom, and which particular aspects can increase patients’ adherence.

## Introduction

Substantial evidence shows that depression among individuals with diabetes is associated with poorer diabetes outcomes [1] and higher levels of diabetes distress (emotional burdens, stresses, and worries associated with diabetes) [2]. When depressed, patients with diabetes show higher frequency of hypoglycemia and higher levels of glycosylated hemoglobin, their risk of developing diabetes-related complications increases [3-5]. They also show poorer adherence to self-care regimens, particularly to medications, diet, and exercise [6-7]. Congruently, diabetes comorbid depression is associated with reduced work productivity [8], reduced quality of life [9], increased medical symptom burden [10], increased functional disability [11], more health care service utilization and costs [12-13], and higher risk of mortality [14-15]. Considering that depression is twice as prevalent among people with diabetes [16], there is no doubt that interventions for depression in individuals with diabetes are crucial for both medical and economic reasons.

Fortunately, there are depression treatments for patients with diabetes [17]. Psychotherapeutic interventions, often combined with a diabetes self-management intervention, have significant effects on depressive symptoms and glycemic control [18-19]. Routine assessment, screening, and treatment of depression in patients with diabetes are recommended in clinical practice guidelines [20]. However, despite these recommendations, depression is both underrecognized and undertreated; in routine care for diabetes, depression remains untreated in 50% of patients [1]. Recently, faced with the need to enhance reach [21], some treatments have resorted to the internet and technological devices as delivery formats. Particularly interesting are interventions requiring considerably less interaction time with the therapist than face-to-face psychotherapy (guided self-help approach) or even no interaction at all (unguided self-help approach). These treatments hold promise for low-cost interventions with positive results [22-23]. They may be particularly beneficial to overcome logistical and financial obstacles burdening both health care providers and patients [24-25]. Other advantages of such interventions are flexible usage not constrained by time and place; a high level of anonymity and privacy; standardized contents, and easy translatability and cultural adaptability [26]. In the general population, effective Web-based interventions and other computerized psychological treatments for depression have been designed and tested in research and slowly but gradually in clinical settings [27]. Moreover, internet-supported therapy for depression with a guided self-help approach has proved to generate the same benefits as face-to-face therapy [28].

In this review, we identified Web-based interventions for depression in individuals with diabetes, addressed those interventions’ efficacy, addressed differences and similarities in interventions’ characteristics and study designs, and discussed studies’ procedures and findings in light of evidence from a wider range of interventions for depression and diabetes. Because psychological intervention studies are very often clinically and methodologically diverse [29], we hope that in the future, our review will be helpful for all researchers and clinicians who are willing to design Web-based interventions for depression in individuals with diabetes.

## Methods

### Inclusion and Exclusion Criteria

Eligible studies had to be published in English or Spanish in a peer-reviewed journal between 1990 (coinciding with introduction of the World Wide Web in 1991) and 2017.

#### Participants

Studies had to target adult participants (18 years or older) with a primary diagnosis of diabetes and comorbid depression. Depression was defined according to diagnostic criteria (*Diagnostic and Statistical Manual of Psychiatric Disorders*) or depressive symptomatology (on a validated self-report or clinician measure).

#### Web-Based Interventions

The examined Web-based interventions required the following components: program content (ie, psychoeducation and skills training guided by psychological theory); multimedia; provision of Web-based activities; and a guided or unguided self-help approach. Eligible interventions had to target depression symptomatology with the specific intent of producing emotional, behavioral, and cognitive change.

#### Study Design

Intervention studies with a repeated measures design, including randomized controlled trials (RCTs) and quasi experimental studies, were eligible.

### Search Strategy

A literature search was conducted in the PsycINFO and MEDLINE electronic databases with the following keywords: diabetes, depression, Web-based, computer-based, internet- based, online, and psychological intervention.

### Data Extraction

The following data were extracted from each study: study characteristics (eg, type of study, sample size, measures); participants’ compliance (eg, dropout percentage); intervention efficacy (eg, between-group effect size in depression and diabetes-related measures); intervention characteristics (eg, delivery mode, psychotherapeutic approach, and research design); sample characteristics (eg, sample size and medical diagnosis); and treatment characteristics (eg, delivery format, therapeutic approach, therapist and peers support, and adherence management). Intervention characteristics sometimes were extracted from the study protocol paper.

Because of the small number of studies and their heterogeneity, data extracted were not statistically combined and reanalyzed. Effect sizes are presented as they were extracted from individual papers’ results sections when the between-groups difference was significant; effect size measures were either Cohen *d* or Hedges *g*.

## Results

### Characteristics of Included Studies

Overall, 5 studies were identified [21,30-33] and all were RCTs. A summary of reviewed articles is provided in Table 1. All studies included standardized measures to assess symptoms of depression and diabetes distress. For depression, studies used the Center for Epidemiological Studies-Depression (CES-D) measure [21,30-32] or the Patient Health Questionnaire-9 (PHQ-9) [33]. Depression inclusion criteria were established in 3 studies [21,32,33]. In Bond et al [30] and Cohn et al’s [31] studies, CES-D was employed but with no established cut-off scores; their treatment groups had a mean baseline CES-D score of 12 (SD 10.4) and 16.9 (SD 11.6), respectively; 3 studies added a telephone-administered interview to confirm whether participants met a major depression episode’s diagnostic criteria [21,32,33]. Newby et al [33] excluded participants with a severe profile (PHQ-9>23).

Diabetes distress was assessed with the Problem Areas in Diabetes Questionnaire (PAID) [21,30,32,33] or the Diabetes Distress Scale (DDS) [31]. All studies added at least one of the following diabetes-related measures: glycosylated hemoglobin [21,33], diabetes self-management [31,32], diabetes empowerment [30], diabetes acceptance [32], and diabetes social support [30].

In addition, 2 studies also assessed the following secondary psychological outcomes [31,33]: anxiety, psychological distress, positive and negative affect, and well-being. Only Nobis et al [32] and Newby et al [33] included process evaluation by expectancy of benefit and intervention satisfaction. All measures were administered online.

### Participants’ Compliance

The percentage of enrolled participants who dropped out (treatment dropout) varied among identified studies: 41.6% (52/125) [21]; 34% (14/41) [33]; 24.0% (31/129) [32]; and 13% (4/29) [31]. Bond et al [30] did not report a treatment dropout rate.

### Intervention Efficacy

Overall, 4 studies found significant reduction in depression scores in the intervention condition compared with control (effect size range 0.29-0.89 for intended-to-treat analyses and 0.70-1.00 for per protocol analyses). See Table 2 for results obtained from the study. Newby et al [33] found that the within-group effects for the intervention group (*g*=1.90) persisted at the 3-month follow-up. Cohn et al’s [31] study showed a reduction in depression scores in the intervention condition compared with the control, although it was not significant (*P*=.05), and found no impact in any other measures.

Significant reduction in diabetes distress was shown in 4 studies (effect size range 0.58-0.80). Newby et al [33] reported that within-group effects for the intervention group (*g*=1.18) persisted at the 3-month follow-up. Positive effects were also found in diabetes social support [30] and diabetes acceptance [32]. Newby et al [33] found moderate positive differences for generalized anxiety and mental well-being that persisted at the 3-month follow-up but failed to find differences in physical well-being and somatic symptom severity. No significant differences were found for glycosylated hemoglobin [21,33] or diabetes self-management [32].

**Table 1:** Summary of studies included in this review.

Lead author (year)	Approach (DM^a^ specific)	Depression criteria	DM type (Age target)	Participants, n	Control	Intervention length	Postassessment
Bond (2010) [30]	CBT^b^ (yes)	N/A^c^	Not reported by authors (older adults)	62	Wait list	6 mo (nonmodular)	6 mo after baseline
van Bastelaar (2011) [21]	CBT (yes)	CES-D^d^>16	I and II (adults)	255	Wait list	8 modules (1 per wk)	1 mo follow-up
Cohn (2014) [31]	Positive psychology (no)	N/A	II (adults)	53	Wait list with emotion reporting	5 modules (1 per wk)	1 wk after the final module
Nobis (2015) [32]	CBT (yes)	CES-D≥23	I and II (adults)	260	Access to unguided Web-based psychoeducation	6-8 modules (1 per wk) + booster session	8 wk after randomization
Newby (2017) [33]	CBT (no)	Patient Health Questionnaire-9>5	I and II (adults)	90	Treatment as usual	6 modules (10 wk, 5 d minimum between)	1 wk after module 6 (or wk 10) 3 mo follow-up for intervention group only

^a^DM: diabetes mellitus.

^b^CBT: cognitive behavioral therapy.

^c^Not applicable.

^d^CES-D: Center for Epidemiological Studies-Depression.

**Table 2:** Results by intervention: Outcome measures, analysis, and effect sizes.

Lead author (year) and outcome measure		Analysis	Effect size
**Bond (2010) [30]**			
	Center for Epidemiological Studies-Depression	Not reported	*d*^a^=0.7
	Problem Areas in Diabetes Questionnaire	Not reported	*d*=0.6
	Diabetes Social Support Scale	Not reported	*d*=1.0
	Diabetes Empowerment Scale	Not reported	*d*=0.7
**van Bastelaar (2011) [21]**			
	Center for Epidemiological Studies-Depression	Intended-to-treat	*d*=0.29
	Center for Epidemiological Studies-Depression	Per protocol	*d*=0.70
	Problem Areas in Diabetes Questionnaire	Per protocol	*d*=0.58
	Glycosylated hemoglobin	Intended-to-treat	—^b^
**Cohn (2014) [31]**			
	Center for Epidemiological Studies-Depression	Per protocol	—
	Perceived Stress Scale	Per protocol	—
	Differential Emotions Scale	Per protocol	—
	Confidence in Diabetes Self-Care Scale	Per protocol	—
	Diabetes Distress Scale	Per protocol	—
**Nobis (2015) [32]**			
	Center for Epidemiological Studies-Depression	Intended-to-treat	*d*=0.89
	Center for Epidemiological Studies-Depression	Per protocol	*d*=1.00
	Hospital Anxiety and Depression Scale-Depression	Intended-to-treat	*d*=0.82
	Problem Areas in Diabetes Questionnaire	Intended-to-treat	*d*=0.58
	Acceptance and Action Diabetes Questionnaire	Intended-to-treat	*d*=0.22
	Diabetes Self-Management Questionnaire	Intended-to-treat	*d*=0.07
**Newby (2017) [33]**			
	Patient Health Questionnaire-9	Intended-to-treat	*g*^c^=0.78
	Problem Areas in Diabetes Questionnaire	Intended-to-treat	*g*=0.80
	Kessler Psychological Distress Scale	Intended-to-treat	*g*=1.06
	Generalized Anxiety Disorder 7-item	Intended-to-treat	*g*=0.72
	Glycosylated hemoglobin	Intended-to-treat	—
	Short form 12-item scale of mental well-being	Intended-to-treat	*g*=−0.66
	Short form 12-item scale of physical well-being	Intended-to-treat	—
	Patient Health Questionnaire physical symptoms module for somatic symptom severity	Intended-to-treat	—

^a^Cohen *d*.

^b^No significance.

^c^Hedges *g*.

### Intervention Characteristics

#### Therapeutic Approach and Delivery Mode

Interventions had a cognitive behavioral therapy (CBT) [21,30,32,33] or a positive psychology [31] psychotherapeutic approach; 3 focused on relevant diabetes-specific topics [21,30,32], whereas the others used generic depression interventions. Interventions aimed to promote different skills. The amount of skills grew proportional to the number of modules presented. The most used topics were psychoeducation, cognitive restructuring, behavioral activation, coping with worries and anxiety, communication and assertiveness, problem solving, and stress management (including breathing and relaxation techniques); 2 interventions addressed relapse prevention [21,34].

**Table 3:** Participants’ activities, clinician-patient communication, and adherence management by intervention.

Lead author (year)	Participant activities	Clinician-assisted, professional	Clinician-patient communication		Adherence management
			Synchronous	Asynchronous	
Bond (2010) [30]	Weekly discussion group & DM^a^ self-management diary	Yes (nurse or psychologist or social worker)	Instant messaging, Web-based educational discussion group	Email and bulletin board	Not reported
van Bastelaar (2011) [21]	Homework	Yes (psychologist)	N/A^b^	Semistandardized feedback on homework assignments (CNE^c^)	Message or email: Homework not received
Cohn (2014) [31]	Homework & mood or DM self-management diary	No	N/A	N/A	Paid for: reports, questionnaires and study completion
Nobis (2015) [32]	Homework & mood diary	Yes (psychologist)	N/A	Email: personalized feedback on homework assignments	Automated daily SMS^d^ text messaging on mobile phone: reminders or motivational Email or phone call: no logging
Newby (2017) [33]	Homework	Yes (psychologist or psychiatrist)	Phone call: patient request or deterioration	Feedback on homework assignments (CNE)	Automated email: reminders or congratulation or no logging Phone call: no logging

^a^DM: diabetes mellitus.

^b^Not applicable.

^c^CNE: channel not specified.

^d^SMS: short message service.

Overall, 4 interventions were distributed in modules. The amount of modules ranged from 5 to 8, and they were delivered with a minimum lapse of 5-7 days between sessions. To strengthen participants’ acquired skills, one study had an optional reinforcing module (“Booster session”) 4 weeks after finishing the intervention [32]. The nonmodular intervention comprised free access to a website with different resources for 6 months with weekly Web-based discussion groups [30].

All interventions provided lesson reinforcement activities and progress tracking; 4 used homework assignments to encourage patients to apply the learned skills in daily practice [21,31,32,34] and 2 added mood, thought, behavior, and diabetes self-management reporting [30,31].

#### Support

##### From Clinician

The identified interventions had important differences concerning therapist support, ranging from none [31] to highly individualized email or phone contact [32]. Bond et al [30] added a weekly discussion group delivered by a Web-based communication forum using MSN Messenger. See Table 3 for therapist support by intervention. Only one study reported clinician time spent per participant; Newby et al [33] reported that the clinician spent, on average, 27 minutes per participant for email and telephone contact over the course of the intervention.

##### From Peers

Only Bond et al’s [30] intervention included contact with peers. This contact was both synchronous and asynchronous by instant short message service (SMS) text messaging and email. Interactions were participant-generated and not moderated by any study personnel.

#### Adherence Management

Two interventions incorporated automated emails or mobile SMS text messaging (integrating mobile phone support) with reminders, motivational statements, and congratulations for finishing a module [32,33]. Three addressed no logging into the website or no homework received by email (mainly standardized) or phone call [21,32,34]. Cohn et al’s [31] intervention used payment as a motivation strategy; they paid US $1 for each daily report completed, US $20 for completing final questionnaires and the phone interview, and US $20 if participants completed the study within 75 days with reports on at least 75% of all days. See Table 3 for adherence management by intervention.

## Discussion

### Main Results and Comparisons with Previous Work

This review aimed to identify Web-based interventions for depression in individuals with diabetes and to discuss these studies’ procedures and findings in light of evidence drawn from a wider range of interventions for depression and diabetes. Overall, 5 studies met the inclusion criteria.

#### Studies’ Characteristics

All studies were RCTs, a rigorous method proven to provide critical evidence for psychological interventions’ efficacy [35]. However, most studies used a wait list as control, and literature shows than this control is more likely than many other control conditions to produce strong effect size [36]. Only one study provided an active control intended to match the intervention’s nonspecific factors [32]. Future research should include active controls matched as closely as possible with the intervention under research but excluding its “active ingredient(s)” [37]. Because no-treatment controls are less costly, they can be potentially useful for novel interventions’ first evaluations. When no-treatment controls are implemented, Mohr et al [36] suggest monitoring health and well-being of participants; assessing and monitoring potential threats to internal validity such as expectancies, help-seeking behavior, and other services received; and continuing assessment for patients dropping out of treatment.

Because people with depression are in need of treatment, a long follow-up period can be inappropriate when a wait list is used as a control group. However, for intervention efficacy and cost-effectiveness, assessing whether effects are long lasting is necessary. One option is to conduct within-group analyses, as performed by Newby et al [33].

All studies included standardized measures to assess depression symptoms and diabetes distress. However, there were differences in the inclusion criteria. If depression diagnosis is an inclusion criterion, it may be advisable to use cut-off scores for CES-D and PHQ-9 of ≥23 and ≥13 points, respectively; these have proven to provide an optimal balance between sensitivity and specificity in people with type 2 diabetes [38]. Nonetheless, a diagnostic interview, at least a telephone-administered diagnostic interview, is strongly recommended. This was included in 3 studies [21,32,33]. This option may indeed increase study costs, but it can also improve the precise diagnosis of depression (if diagnosis is an inclusion criterion). An even better scenario consists of a face-to-face diagnostic interview, precluding problems of not seeing the patient. A meta-analysis showed that in both controlled and uncontrolled studies, depression rates are approximately two to 3 times higher in studies that used self-report measures versus face-to-face diagnostic interviews [16]. Using Web-based questionnaires to assess depression symptomatology usually works well, but psychiatric diagnoses cannot be reliably made using self-reports solely. If face-to-face interviews cannot be conducted, a compromise solution could lie in telephone interviews to confirm the diagnosis [39].

For diabetes distress assessment, Schmitt et al’s [40] recent study supported both PAID and DDS as good self-report measures of diabetes distress. They concluded that PAID was significantly more strongly associated with dysfunctional coping styles, quality of life, and depressive symptoms, whether DDS showed significantly stronger associations with diabetes self-care and metabolic outcomes; therefore, its selection should be based on study objectives. A cut-off criterion’s inclusion concerning these measures should be considered, particularly for interventions with diabetes-specific content.

Because of the impact of depression-diabetes comorbidity on diabetes self-management and glycemic control [3,4,5], inclusion of these variables is desirable. Van Bastelaar et al [21] and Newby et al [33] did not find an effect on glycosylated hemoglobin, but in their studies, participants’ diabetes was relatively well controlled, despite comorbid depression and high levels of diabetes distress. Face-to-face treatments for depression have shown mixed results for glycosylated hemoglobin outcomes [41] so that more evidence is needed to clarify this relationship, including its moderators and mediators. Adding recurrent glycemia monitoring can probably foster a broader understanding of the intervention effect and its relation with CBT-targets (cognition, emotion, and behavior). Newer Web platforms include emotion, thoughts and behavior registers, and sometimes graphics [42]. Glycemia data graphics could be easily integrated. This would also function as a resource itself, providing patients feedback about the relationship between depression symptomatology and glycemic control.

Assessment of other psychological outcomes (eg, anxiety) and process evaluation (eg, satisfaction with the intervention) can provide a more comprehensive assessment of the intervention’s effects, identify individuals most likely to benefit, and identify adherence-related dimensions. More recent studies have tended to include these variables [31-33]. Additionally, recruitment strategies should be taken into account (eg, online) because they can lead to selection bias. The use of questionnaires to assess reasons for participating, expectancies, credibility, and patient satisfaction with the intervention are strongly suggested [32].

#### Participants’ Compliance and Treatment Efficacy

As noted in this review, Web-based interventions often suffer from nonadherence. A systematic review of 83 Web-based interventions on lifestyle, chronic disease, and mental health (with and without therapist support) found that, on average, approximately 50% of participants adhere fully to an intervention [43]. A meta-analysis that compared adherence to Web-based and face-to-face CBT for depression (although not in a single trial) found significant differences in the percentage of treatment completers with 65% and 84%, respectively [44]. Nonetheless, authors of the meta-analysis found that in the Web-based CBT, participants completed, on average, 80% of their treatments; this does not differ significantly from the rates observed in face-to-face CBT groups. They suggest that future studies should include more detailed information on adherence, preferably both the number of completers and average number of sessions completed, and should search for factors that can explain adherence and participants’ reasons for dropping out.

Interventions’ impact on depressive symptoms are consistent with previous research on Web-based depression treatments in the general population with meta-analyses showing an effect size of *d*=0.4 [27] and *d*=0.56 [45] that increases to *d*=0.61 and *d*=1.35, respectively, when supported by a therapist. This lends support to the notion that Web-based interventions have potential as a psychology service delivery option for individuals with diabetes and depression.

#### Interventions’ Characteristics

Overall, 4 interventions had a CBT only approach. Face-to-face CBT is the most extensively researched psychotherapeutic treatment for depression [46] and has shown to be effective in depression symptomatology [47] and glycemic control [48] in individuals with diabetes. CBT is also the most commonly used approach in Web-based depression interventions [45,49,50]. Furthermore, Web-based guided self-help CBT is the only approach that has been directly compared with face-to-face CBT with reported effects being similar [28]. However, all interventions are multicomponent with a number of hypothesized mechanisms (eg, behavioral activation and cognitive restructuring); therefore, determining which aspects contribute the most in psychological outcome measures is not possible. The combination of approaches may not be suggested because it makes drawing clear conclusions regarding effective ingredients even more difficult. Future studies must elucidate which skills should be promoted for stronger effect in depression and what mechanisms of change are.

The need of diabetes-specific content should be studied. An aspect that can contribute to understanding of the nondiabetic specific versus diabetic-specific debate is probably related to the presence or absence of diabetes distress. Both syndromes are closely related, but also independent, and they can co-occur or not [51]. Snoek et al [51] advanced the following 3 possible combinations of depression and diabetes among diabetic patients: with distress, but no depression; with depression, but no distress; and no depression or distress. They propose that the first 2 are more are likely to benefit from diabetes-specific depression treatment modalities. However, Newby et al’s [33] nondiabetes-specific intervention showed large effects on depression *and* diabetes distress, whereas it showed no significant effects on glycemic control. In the past, both face-to-face health care and digital interventions have tended to focus on either depression or diabetes alone, despite their co-occurrence and similar behavioral treatment strategies that may call for a more holistic approach [52]. A review of Web-based interventions for comorbid depression and chronic illness showed that participants valued psychoeducation with illness-specific examples [53]. Perhaps an intervention for depression *and* diabetes, instead of *in* individuals with diabetes, may contribute to address health in a more holistic way.

Weekly modules tend to mimic face-to-face therapy sessions’ frequency. Approaches like CBT normally prioritize short-term care; therefore, the average number of sessions for depression face-to-face treatment is approximately 13 [54] with brief forms containing less than 8 sessions. The ideal number of modules remains unclear, mainly because when interventions with different numbers of modules are compared, they differ in other unmeasured key variables such as the modules’ content; thus, whether the impact on depression symptomatology is because of the number of modules alone remains uncertain [54]. On the other hand, evaluating the effect of the patient choosing which modules to complete and proving recommendations to participants on which modules are more suitable for them from the assessment upon registration would be interesting [42].

All interventions provided lesson reinforcement activities or progress tracking. Homework is important for helping patients to apply skills learned during sessions to various and multiple situations that arise in everyday life [55]. In face-to-face CBT for depression, the assignment of homework and homework compliance show significantly better outcomes than therapy consisting only of work during the session [54]. On the other hand, inclusion of regularly self-administered questionnaires or reports may have benefits by allowing both patients and therapists to monitor progress and deterioration of depression.

#### Support

Consistent with meta-analyses, a Web-based intervention’s effect on depression is greater when the intervention includes therapist assistance or guidance with patients’ compliance being higher [27,45]. Communication between patient and therapist in the identified studies was mainly asynchronous (personalized or semipersonalized), providing feedback on homework and other issues. Synchronous communication in the selected studies was used for adherence management after nonresponse to asynchronous strategies or for urgent cases like deterioration. Interestingly, a meta-analysis on Web-based depression interventions showed that studies providing asynchronous support yielded greater effects than studies providing synchronous support [45] perhaps because of the benefits associated with asynchronous communication such as disinhibition and more time to reflect and compose one’s responses [56]. A study that compared 2 groups allocated to a Web-based CBT for depression with therapist guidance either by telephone calls or email correspondence showed significant and large symptom reductions in both groups with no significant differences between them [57]. There was no between-group difference in client-rated therapeutic alliance or treatment engagement. However, more research is needed to determine how the content, length, and frequency of therapists’ feedback can affect outcomes in guided self-help treatments [58].

Newby et al’s [33] intervention established a clinician’s email or phone call when participants requested contact or had a depression or distress score indicating deterioration of their condition. A review of Web-based programs for depression currently available in English showed that 62% (20/32) had a crisis link defined as email addresses, phone numbers, or hotlines connected to distress centers providing counseling services to at-risk users [49]. Because of the depression’s oscillating course, risk of deterioration or moments of crisis always exist; therefore, detecting them in time and determining courses of action are important.

Only one intervention incorporated peer support [30]. This reflects the small number of Web-based interventions that offer such support. A Web-based intervention for diabetic support showed that online peer support was a successful approach [42], but in interventions for depression, evidence is limited and inconsistent [59]. Future studies should bring new data to this subject.

#### Adherence Management

Considering that adherence is problematic in Web-based interventions [60,61] for many depressed people [45,62] and for many people with diabetes [63] and that it is associated with treatment effectiveness [64,65], the need exists to develop and evaluate ways to increase intervention adherence. This review and the literature have shown that frequent automated reminders via email or SMS text messaging can positively influence adherence [43]. However, through studies that compare strategies, it remains necessary to determine which adherence management techniques are more effective.

### Limitations

Caution is needed when drawing conclusions from efficacy results exposed by this review. In most studies, participants were well educated overall with relatively well controlled diabetes. In 2 studies [30,31], the number of participants was relatively small (25 and 31 in treatment groups), which also affects the generalizability of results. Different measures and cut-off criteria for depressive symptomatology and multicomponent interventions make comparing studies’ results difficult. Finally, as mentioned above, the recruiting strategy could have led to selection bias in some cases.

This review has some limitations. We found only 5 studies that met our criteria; therefore, caution is needed when trying to generalize results. These findings may be affected by publication bias with a tendency for academic journals to publish significant findings. Because we restricted our literature search to articles written in English or Spanish, we might have missed studies eligible for inclusion but published in other languages.

### Conclusions

In summary, we are optimistic about Web-based interventions for depression in people with diabetes. Our review and the literature support the idea that with the inclusion of specific features (such as some therapist support), these interventions are effective. They may enhance the therapy’s reach and decrease both patient and health services costs by not only engaging in a less expensive, more accessible treatment but also preventing diabetes complications and depression deterioration. Upcoming research should continue contributing evidence on why these interventions are effective, for whom, and which aspects can increase patient adherence. We hope that, in the future, our review will be helpful for all researchers and clinicians willing to design and use Web-based interventions for depression in individuals with diabetes.

## Abbreviations

CBT: cognitive behavioral therapy
CES-D: Center for Epidemiological Studies-Depression
DDS: Diabetes Distress Scale
PAID: Problem Areas in Diabetes Questionnaire
PHQ-9: Patient Health Questionnaire-9
RCT: randomized controlled trial
SMS: short message service
